# Comparison of diestrus duration and temporal gene expression patterns in vaginal smear samples from pseudopregnant rats induced by sonic vibration or mating with vasectomized males

**DOI:** 10.1186/s42826-026-00290-w

**Published:** 2026-08-18

**Authors:** Sungok Hong, Kyung Soo Kang, Hongduk Kim, Ran Kim, Kangyoun Kim, Yoodam Ha, NaHyun Kim, Minjae Kim, Jun-Won Yun, Tae Min Kim

**Affiliations:** 1 Institutes of Green-Bio Science and Technology, Seoul National University, Pyeongchang, Gangwon-do 25354 Republic of Korea; 2 Department of Bio Life Science, Life & Environment Field, Shingu University, Seongnam, 13174 Republic of Korea; 3 Graduate School of International Agricultural Technology, Seoul National University, Pyeongchang, Gangwon-do 25354 Republic of Korea; 4 Laboratory of Veterinary Toxicology, College of Veterinary Medicine and Research Institute for Veterinary Science, Seoul National University, Seoul, 08826 Republic of Korea; 5 Department of Agricultural Biotechnology, College of Agriculture and Life Sciences, Seoul National University, Seoul, 08826 Republic of Korea

**Keywords:** Rat, Pseudopregnancy, Sonic vibration, Artificial reproductive technology, Vaginal cytology

## Abstract

**Background:**

Induction of pseudopregnancy is a prerequisite for successful embryo transfer in rats. Conventionally, pseudopregnancy is induced by mating females with vasectomized males. Although effective, this method requires surgical preparation and maintenance of sterile males and may be affected by variability in mating performance. Artificial induction using sonic vibration has been proposed as a practical alternative; however, its comparability to the conventional method regarding the duration of the pseudopregnant state and temporal changes in the genes in the vaginal tissue has not been investigated.

**Results:**

Pseudopregnancy was induced in Sprague-Dawley females either using sonic vibration or cohabitation with vasectomized males. The duration of pseudopregnancy was monitored via vaginal cytology. Vaginal smear-derived samples were collected during the first diestrus, middle diestrus, and the diestrus immediately before proestrus. Subsequently, the temporal expression of genes associated with estrus cycles (Filaggrin, Progesterone Receptor, Krt14, Il-1β, Zonula occludens-1, Occludin, Claudin1, Cdh1) was analyzed using quantitative real-time polymerase chain reaction. Generally, the duration of diestrus was longer when pseudopregnancy was induced by artificial stimulation (12–22 days) compared with that induced by a vasectomized male (10–12 days). Overall, the gene expression patterns in vaginal smear-derived samples were broadly similar between females subjected to sonic vibration and those mated with vasectomized males.

**Conclusions:**

Sonic vibration appeared to maintain a pseudopregnancy-like diestrus period for a longer duration than conventional mating-based methods. Changes in mRNA expression in the vaginal smear samples during the diestrus stage were similar between the two methods. Although further validation, including hormonal and uterine assessments, is needed, sonic vibration may represent a useful alternative method for reproductive engineering in rats by reducing the need for vasectomized males and simplifying colony management.

**Supplementary Information:**

The online version contains supplementary material available at 10.1186/s42826-026-00290-w.

## Background

Induction of pseudopregnancy is essential for successful embryo transfer in rats [1–3], commonly achieved by mating recipient females with vasectomized males [3, 4]. However, this method requires continuous preparation, surgery, maintenance, and monitoring of vasectomized males, increasing animal use, housing space, labor, and costs [1, 5].

Sonic vibration presents a practical alternative and induces pseudopregnancy in rats, supporting the development of transferred embryos into live offspring [1, 2]. Artificial stimulation may reduce dependence on vasectomized males and contribute to the refinement of assisted reproductive techniques [3]. However, the biological comparability of pseudopregnancy induced by sonic vibration with that induced by vasectomized male mating has not been sufficiently evaluated.

The vaginal epithelium, responsive to hormones, undergoes marked structural, cytological, and molecular changes throughout the estrous cycle [6–9]. Thus, vaginal smear cytology is a reliable and non-invasive indicator of reproductive stage in rodents [6, 7]. The vaginal epithelium exhibits hormone-dependent regulation of progesterone receptors and barrier-associated molecules [10–13]. Diestrus is particularly relevant in pseudo-pregnancy studies because it reflects the progesterone-dominant state associated with luteal maintenance [14].

Herein, we compared diestrus duration in female rats with pseudopregnancy induced either by sonic vibration or mating with vasectomized males. We analyzed the temporal gene expression patterns in vaginal smear-derived samples collected during early, middle, and late diestrus using quantitative real-time polymerase chain reaction (qPCR). We hypothesized that if sonic vibration induces a pseudopregnant state comparable to that produced by conventional mating, the duration of pseudopregnancy and vaginal epithelial gene expression patterns would be similar between the two groups. Demonstrating such comparability supports sonic vibration as a practical and reproducible alternative to conventional mating-based pseudopregnancy induction.

## Main text

### Animals

All animal experiments were approved by the Institutional Animal Care and Use Committee of the Seoul National University (no. SNU-251215-3). All animals were purchased from Koatech (Pyeongtaek, Korea) and maintained under specific-pathogen-free conditions, controlled environmental conditions with a 12 h light/dark cycle, and free access to food and water. For pseudopregnancy induction, seven and nine female 12-week-old Sprague-Dawley (SD) rats were mated with vasectomized males and subjected to sonic vibrations, respectively. Seven male 13-week-old SD rats were used for bilateral vasectomy. Upon reappearance of proestrus/estrus, female rats were euthanized using CO_2_ asphyxiation.

### Vasectomy

Thirteen-week-old male SD rats were anesthetized and subjected to bilateral vasectomy, as previously described [15]. Postoperative analgesia was administered subcutaneously with carprofen (5 mg/kg) once daily for 3 days. The animals were allowed to recover for two weeks before use. All males were kept individually for 10 days before mating with females.

### Induction of pseudopregnancy

**Fig. 1:**
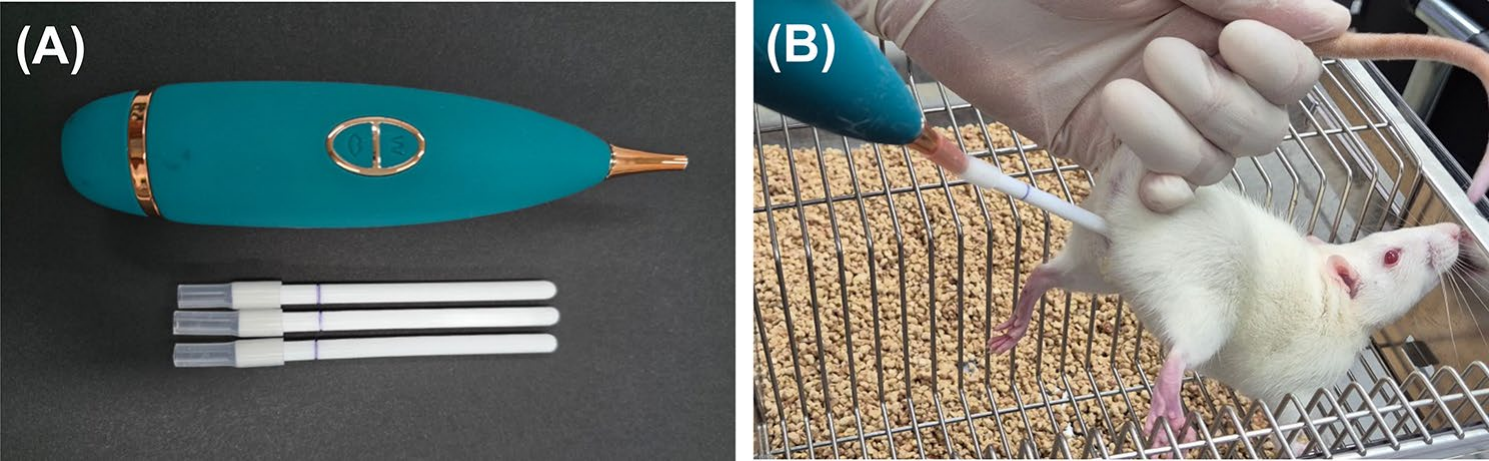
Sonic vibration. (**A**) Configuration of sonic vibrator used for artificial stimulation. (**B**) Intravaginal sonic vibration in a female rat

To synchronize the estrus cycle in each animal, we intraperitoneally administered luteinizing hormone-releasing hormone (200 μg, Sigma-Aldrich, MO, USA) to females. Vaginal cytology was performed 4 days later. Animals confirmed to be in proestrus were subjected to either artificial stimulation using sonic vibration or co-housed overnight with a vasectomized male for pseudopregnancy induction. For artificial stimulation, sonic stimulation was performed using a custom-built vibrator with a rated output of 0.75 W and a frequency of 20,000 cycles per minute (Fig. 1A). The stimulator was then attached to a fluororesin probe (4 mm diameter, 7 cm length; Fig. 1A). The probe was carefully introduced into the vagina until it contacted the cervical canal (Fig. 1B), after which vibration was conducted twice for 30 s with 30 s interval [2]. The day on which artificial stimulation or mating was performed was designated as day 0 of the pseudopregnancy.

### Evaluation of pseudopregnancy

Vaginal smear cytology was monitored every 1 or 2 days before the animals returned to proestrus. Estrous cycle stages were determined based on predominant cell populations, including nucleated epithelial cells, cornified epithelial cells, and leukocytes [7]. The pseudopregnancy duration was defined as the continuous period during which the animals remained in diestrus following induction, until the reappearance of proestrus/estrus cytology [1, 2].

### Experimental sampling points

Vaginal smear-derived samples were collected at three defined time points during pseudopregnancy (1): the first day of diestrus after induction (2), the mid-diestrus stage, and (3) diestrus immediately before proestrus/estrus.

### Vaginal smear collection and preparation

Collection and crystal violet staining of vaginal tissues were conducted as previously described [7, 16] (Additional file 1).

### Tissue collection, RNA extraction, cDNA synthesis, and qPCR

After slide application, the remaining cotton swab was immersed in a 1.7-mL tube and manually agitated for 10 s in ice-cold phosphate-buffered saline. After centrifugation at 300 × *g* for 1 min, total RNA was extracted using TRIzol® (Thermo Fisher Scientific, Carlsbad, CA, USA) following the manufacturer’s instructions. cDNA was synthesized using 500–1,000 ng of total RNA using a cDNA synthesis kit (Philekorea, Daejeon, Korea). qPCR was performed using an Accupower® 2X GreenStar qPCR Master Mix (Bioneer, Daejeon, Korea) in a CFX96 Touch Real-Time PCR Detection System (Bio-Rad Laboratories, Inc., Hercules, CA, USA). Gene expression was normalized against *Gapdh*, and the relative expression was analyzed using the 2^-ΔΔCt^ method [17]. The primer sequences are listed in Additional file 2.

### Statistical analysis

Data are presented as mean ± standard error of the mean. For gene expression analyses, differences among sampling time points were evaluated using a two-way analysis of variance, followed by Bonferroni’s test. Relative mRNA expression was calculated using the 2^-ΔΔCt^ method and expressed as fold-change values. Statistical analyses of qPCR data were performed using ΔCt values [17]. Normality of model residuals was assessed using the Shapiro–Wilk test, and homoscedasticity was evaluated using residual and homoscedasticity plots together with Spearman’s test for heteroscedasticity. Statistical significance was set at *p* < 0.05. All analyses were performed using GraphPad Prism 11.0.

## Results

### Estrous stage changes after mating with vasectomized male rats

**Fig. 2:**
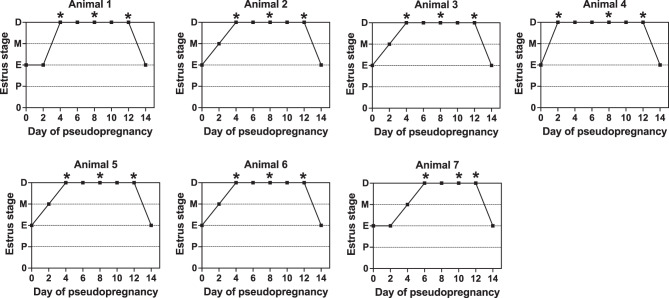
The estrus cycle stage of female rats after being housed with vasectomized male rats. The stages are proestrus (**P**), estrus (**E**), metestrus (**M**), and diestrus (**D**). D0 denotes 96 hours after LH-RH administration, when animals were mated with vasectomized male. (*) denotes time point when RNA was extracted for qPCR

Representative vaginal smear images for each estrous cycle stage are shown in Additional file 1. The estrous stages of female rats after mating with vasectomized male rats are summarized in Fig. 2. On D0, defined as 96 h after administration and the time point of mating with vasectomized males, all animals were in the estrus stage. After mating with vasectomized males, the animals rapidly shifted toward metestrus/diestrus by D4, and this diestrus stage was maintained up to D10–12.

### Estrus stage changes after artificial stimulation

**Fig. 3:**
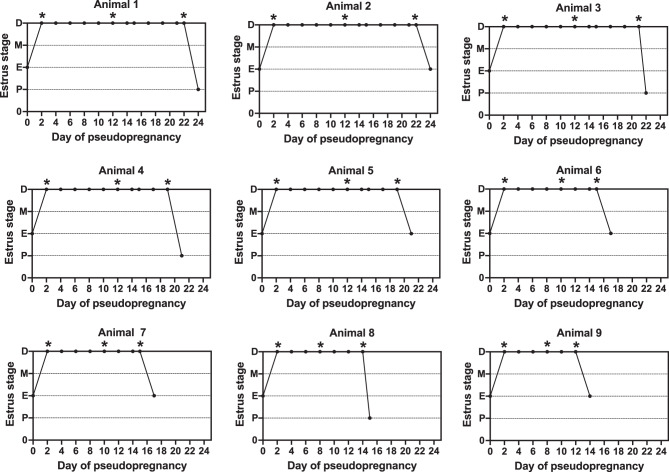
The estrus stage of female rats after artificial stimulation. The stages are proestrus (**P**), estrus (**E**), metestrus (**M**), and diestrus (**D**). D0 denotes 96 hours after LHRH administration, when animals were stimulated via sonic vibration. (*) denotes time point when RNA was extracted for qPCR

The estrous stages of female rats after artificial stimulation are shown in Fig. 3. All animals were in estrus on D0 and shifted to diestrus on D2. The return to proestrus/estrus occurred gradually at later time points ranging from D14 to D24.

### Temporal gene expression changes during early, middle, and late diestrus

**Fig. 4:**
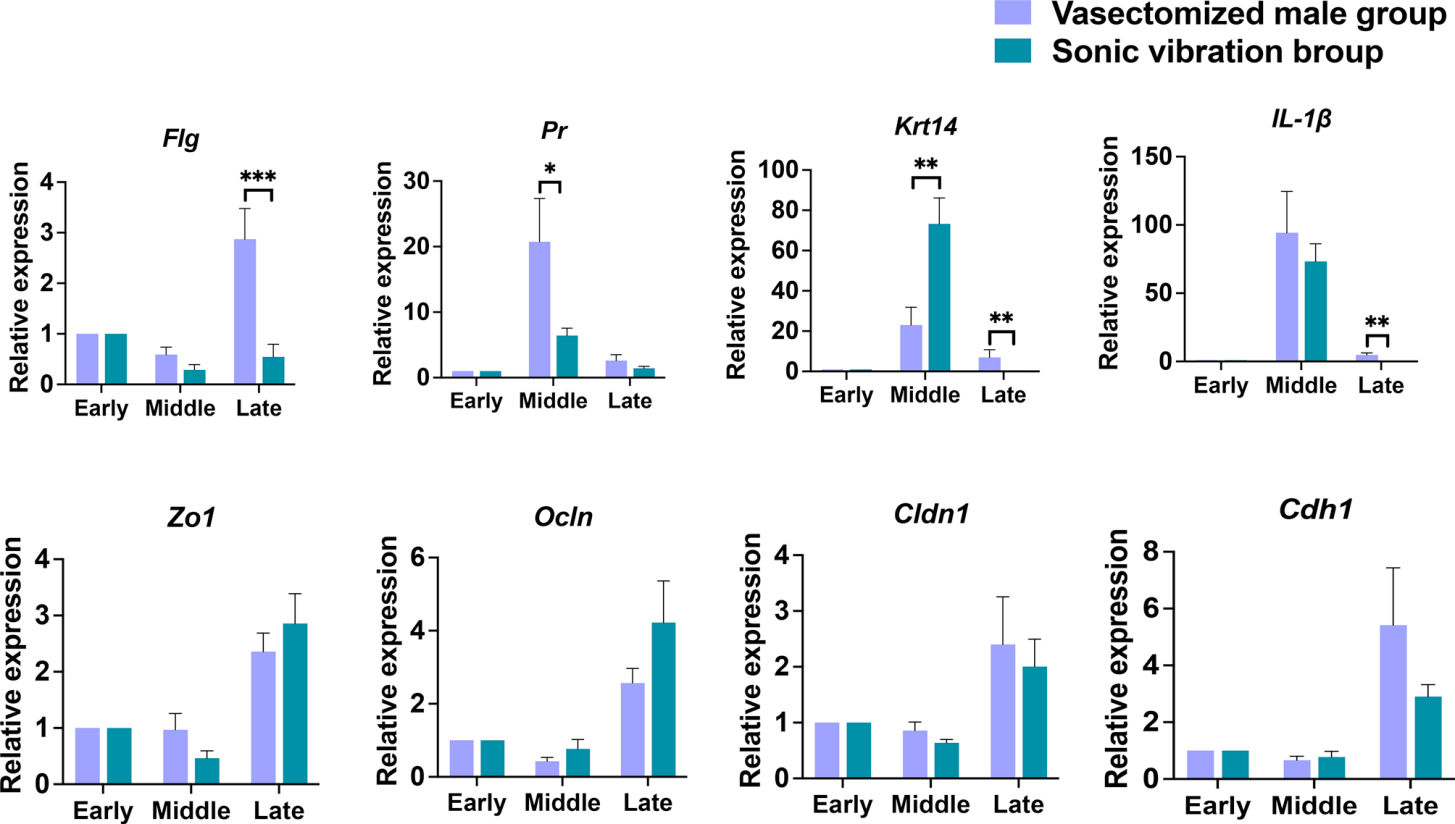
Relative mRNA expression in vaginal smear cells during diestrus after pseudopregnancy induction by two different methods. qPCR analysis was performed using vaginal epithelial cells collected during early, middle, and late diestrus after vasectomized male mating and sonic vibration. Graphs show fold-change values calculated using the 2^-ΔΔCt^ method. Statistical analyses were performed using ΔCt values by two-way ANOVA followed by Bonferroni’s multiple comparisons test. Data are mean ± SEM. **p < 0.01 and ****p < 0.0001

The selected genes were selected to represent biological features of vaginal smear-derived epithelial cells, including steroid hormone responsiveness (*Pr*) [10, 18], epithelial differentiation/cornification (*Flg* and *Krt14*) [19, 20], mucosal inflammation (*Il-1β*) [21], and epithelial junctional integrity (*Zo1, Ocln, Cldn1, and Cdh1*) [10, 22] (Fig. 4). During early diestrus, the expression levels of all examined genes were comparable between the two groups. During late diestrus, *Flg* expression was significantly higher in the vasectomized male group than in the sonic vibration group. During middle diestrus, *Pr* expression was augmented in the vasectomized male group compared with the sonic vibration group, whereas *Krt14* expression was significantly higher in the sonic vibration group. *Il-1β* expression was increased at middle diestrus in both groups, but no significant difference was observed at this stage. Additionally, increased expression of *Krt14* and *Il-1β* was observed in the vasectomized group during late diestrus. The junction-associated genes *Zo1*, *Ocln*, *Cldn1*, and *Cdh1* exhibited stage-related changes, particularly toward late diestrus; however, their levels did not differ between the two groups.

## Discussion

The prolonged diestrus observed after sonic vibration may be related to the nature of the cervicovaginal stimulus. In rats, pseudopregnancy is initiated by mating or mechanically induced cervicovaginal stimulation, which evokes prolactin surges and supports luteal progesterone secretion [15]. A previous study has demonstrated that prolactin surges initiated by cervical stimulation were required for functional transformation and maintenance of the corpus luteum during pseudopregnancy [23]. Therefore, the longer diestrus duration in the sonic vibration group may reflect a more consistent or sufficient cervicovaginal stimulus than that achieved by natural mating with vasectomized males, in which the intensity and number of intromissions can vary among pairs. However, this interpretation remains speculative and should be confirmed by endocrine profiling in future studies.

The 22-day prolonged diestrus/pseudopregnancy-like status observed in this study supassed previous studies. Kaneko et al. have reported diestrus maintenance for at least 14 days post-sonic vibration, whereas subsequent studies mainly addressed embryo transfer outcomes and did not report diestrus maintenance beyond 20 days [1, 2]. The 22-day duration should be regarded as an unusually prolonged. Variations may arise from neuroendocrine response to vaginocervical stimulation. In rats, cervical or vaginal stimulation induces pseudopregnancy through the brain–pituitary–ovary reflex, in which prolactin surge provides luteotropic support to the corpus luteum and maintains progesterone secretion [24, 25]. Thus, a stronger luteotropic response may have resulted in delayed luteolysis and prolonged progesterone-dominant vaginal However, because circulating prolactin, progesterone, and estradiol levels were not measured, prolonged cases should be interpreted cautiously.

The selected qPCR markers should be interpreted as functional indicators of the vaginal epithelial environment during the diestrus-like pseudopregnant state [26]. *Pr* may reflect the steroid-hormone responsiveness of the vaginal epithelium, whereas *Krt14*, a marker of basal epithelial cells, may indicate changes in basal/parabasal epithelial contribution or epithelial turnover during diestrus [18, 19]. Because vaginal smear-derived samples contain mixed epithelial cell populations, this interpretation should be further confirmed using histological or immunohistochemical analyses. *Flg* is related to terminal epithelial differentiation and keratinization, which are prominent cyclic features of the rodent vaginal epithelium. Therefore, changes in *Flg* expression may reflect the extent or timing of progression toward a cornified epithelial phenotype rather than serve as a direct marker of pseudopregnancy. The relatively higher expression of *Pr, Krt14, and Il-1β* during middle diestrus may reflect a transient phase of hormone-responsive epithelial remodeling and mucosal immune surveillance [18, 27, 28]. *Il-1β* is particularly relevant because innate immune mediators in the female reproductive tract are cyclically regulated, and vaginal *IL-1β* varies across the estrous cycle [21, 28]. In contrast, the later increase in *Zo1, Ocln, Cldn1, and Cdh1* suggests progressive reinforcement of epithelial junctional integrity as diestrus proceeds [20]. Although the underlying mechanism remains largely unexplored, this interpretation may be supported by previous work showing that *Zo1, Ocln*, and *cldn1* are expressed in the rat vaginal epithelium and regulated by estrogenic conditions [10]. However, gene expression profiles from these samples should be interpreted with consideration of both epithelial status and smear cell composition because vaginal smears consist primarily of exfoliated epithelial cells but may also contain variable numbers of leukocytes depending on the estrous cycle stage.

This study had several limitations. Histological and immunohistochemical analyses of the vagina, cervix, or uterus could confirm whether tissue-level responses are identical between pseudopregnancies induced by sonic vibration and mating with vasectomized males [14]. Comparing hormone levels during each estrous cycle could reveal the hormonal mechanism involved. This study focused primarily on pseudo-pregnancy duration and vaginal molecular profiles rather than direct reproductive endpoints such as implantation rate and live birth after embryo transfer. Further studies integrating recipient tissue analyses with embryo transfer outcomes in the same experimental settings would strengthen the translational value of this model.

## Conclusions

Artificial stimulation using sonic vibration induced a prolonged diestrus state and resulted in temporal expression patterns of selected vaginal smear-derived genes that partially overlapped with those observed after vasectomized males, supporting sonic vibration as a promising approach for inducing pseudopregnancy in rats, although further data are needed.

## Electronic supplementary material

Below is the link to the electronic supplementary material.

Supplementary Material 1: Representative vaginal smear images illustrating the characteristic cellular composition at each stage of the estrous cycle. (A) Proestrus in which nucleated epithelial cells are predominantly observed. (B) Estrus, characterized by a large number of cornified squamous epithelial cells. (C) Metestrus, in which cornified epithelial cells are accompanied by an increasing number of leukocytes. Arrowheads indicate polymorphonuclear cells. (D) Diestrus, where leukocytes are the major cell population among nucleated epithelial cells (arrows)

Supplementary Material 2: Primer sequences used in this study

## Data Availability

The data used in this study are available from the corresponding author upon reasonable request.

## Abbreviations

ET: Embryo transfer
LH-RH: Luteinizing hormone-releasing hormone
PCR: Polymerase Chain Reaction
Pr: Progesterone receptor
rRNA: ribosomal ribonucleic acid
SD: Sprague-Dawley
SEM: Standard error of the mean
Zo1: Zonula occludens 1
