# Supplementary material for: Comparison of diestrus duration and temporal gene expression patterns in vaginal smear samples from pseudopregnant rats induced by sonic vibration or mating with vasectomized males
Source: Lab Anim Res. 2026 Aug 18;42:28. doi: 10.1186/s42826-026-00290-w (PMC13483139; doi:10.1186/s42826-026-00290-w)
Supplement: Supplementary file 1 [file 42826_2026_290_MOESM1_ESM.pdf]

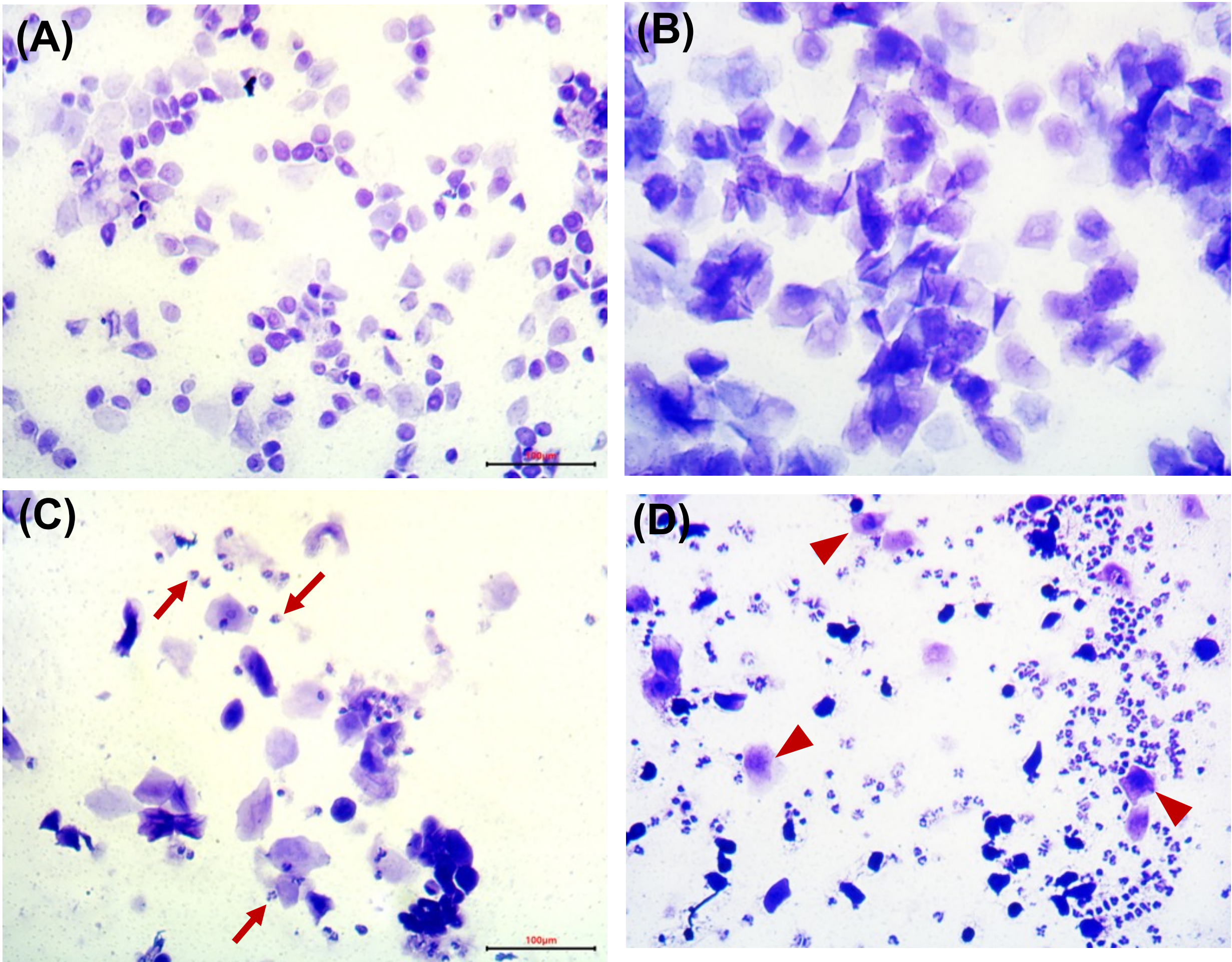

(A)
100μm
(B)
(C)
100μm
(D)
