# Supplementary material for: Comparison of diestrus duration and temporal gene expression patterns in vaginal smear samples from pseudopregnant rats induced by sonic vibration or mating with vasectomized males
Source: Lab Anim Res. 2026 Aug 18;42:28. doi: 10.1186/s42826-026-00290-w (PMC13483139; doi:10.1186/s42826-026-00290-w)
Supplement: Supplementary file 2 [file 42826_2026_290_MOESM2_ESM.docx]

Primer sequences used for qRT-PCR.

| Genes | Forward (5’>3’) | Reverse (5’>3’) |
| --- | --- | --- |
| *Rat 18s rRNA* | GTGGAGCGATTTGTCTGGTT | CGCTGAGCCAGTTCAGTGTA |
| *Rat Zo1* | TTCGCCTGAAACAAACCCAG | CTTGTGATACGTGCGAGGTG |
| *Rat Ocln* | GTGGCTTCCACACTTGCTTG | TGTACCCTCCGTAGCCGTAA |
| *Rat Cdh1* | ATCCTGGCCCTCCTGATTCT | ACTTCCGGTCTGGCATCAAG |
| *Rat Pr* | GACAACACAAAGCCCGACAC | CGGAAACCTGGCAGAGACTT |
| *Rat IL1β* | AAGAATCTATACCTGTCCTGTG | CAAACTCCACTTTGGTCTTG |
| *Rat Flg* | GCCACAAGCATTGACAGCAG | TGCCTCTGGCTTGTTTGGAT |
| *Rat Krt14* | CCACGAGGAGGAAATGGCTT | CAATCTTCGGCATCCTTGCG |
